# Successful Ovariotomy in a Child Four Months Old

**Published:** 1898-03-12

**Authors:** 


### SUCCESSFUL OVARIOTOMY IN A CHILD FOUR MONTHS OLD.

A very remarkable case has been recorded by Mr. D'Arcy Power, in which he removed a large thick-walled cystic ovarian tumour, causing great distension of the abdomen, from a child aged four months. The tumour was attached by a pedicle about the thickness of a slate pencil, which was tied in the usual manner with fine silk, divided, and dropped back into the abdomen. The child was a little collapsed after the operation, but it soon rallied and made a perfectly uneventful recovery, being able to leave the hospital within a fortnight of the operation.

